# Isolation and characterization of bovine herpesvirus 4 (BoHV-4) from a cow affected by post partum metritis and cloning of the genome as a bacterial artificial chromosome

**DOI:** 10.1186/1477-7827-7-83

**Published:** 2009-08-19

**Authors:** Gaetano Donofrio, Valentina Franceschi, Antonio Capocefalo, Sandro Cavirani, Iain Martin Sheldon

**Affiliations:** 1 Dipartimento di Salute Animale, Facoltà di Medicina Veterinaria, Università di Parma, via del Taglio 10, 43100 Parma, Italy; 2 Institute of Life Science, School of Medicine, Swansea University, Singleton Park, Swansea, SA2 8PP, UK

## Abstract

**Background:**

Bovine herpesvirus 4 (BoHV-4) is a gammaherpesvirus with a Worldwide distribution in cattle and is often isolated from the uterus of animals with postpartum metritis or pelvic inflammatory disease. Virus strain adaptation to an organ, tissue or cell type is an important issue for the pathogenesis of disease. To explore the mechanistic role of viral strain variation for uterine disease, the present study aimed to develop a tool enabling precise genetic discrimination between strains of BoHV-4 and to easily manipulate the viral genome.

**Methods:**

A strain of BoHV-4 was isolated from the uterus of a persistently infected cow and designated BoHV-4-U. The authenticity of the isolate was confirmed by RFLP-PCR and sequencing using the TK and IE2 loci as genetic marker regions for the BoHV-4 genome. The isolated genome was cloned as a Bacterial Artificial Chromosome (BAC) and manipulated through recombineering technology

**Results:**

The BoHV-4-U genome was successfully cloned as a BAC, and the stability of the pBAC-BoHV-4-U clone was confirmed over twenty passages, with viral growth similar to the wild type virus. The feasibility of using BoHV-4-U for mutagenesis was demonstrated using the BAC recombineering system.

**Conclusion:**

The analysis of genome strain variation is a key method for investigating genes associated with disease. A resource for dissection of the interactions between BoHV-4 and host endometrial cells was generated by cloning the genome of BoHV-4 as a BAC.

## Background

Uterine infections are important because they disrupt not only the function of the uterus, but also the ovary and the overarching higher control centres in the hypothalamus and pituitary [1]. The inflammatory and immune response to uterine infection compromises animal welfare as well as affecting fertility. Indeed, uterine disease causes infertility during infection and sub-fertility even after successful resolution of the disease. Understanding the mechanisms underlying the effect of microbial infection and the associated immune response on bovine reproduction is important to develop new treatments and disease prevention strategies [2].

Postpartum metritis or pelvic inflammatory disease affects up to 40% of dairy cattle [1]. It is assumed that most uterine disease is of bacterial origin. Virus isolation or serology is rarely considered even though abortion may follow infection with a variety of alpha-, beta- and gammaherpesvirus. Bovine herpesvirus 4 (BoHV-4) is a virus consistently associated with cases of bovine metritis. The first reported isolation of BoHV-4 from a case of bovine metritis was in 1973 [3]. Later several other isolates were obtained from cows with reproductive disorders from several countries, including Italy [4] and India [5]. In Belgium, BoHV-4 seroprevalence was associated with postpartum metritis, and chronic infertility of cattle [6]. Postpartum metritis has also been associated with BoHV-4 in the USA [7,8], Spain [9] and Serbia [10].

BoHV-4 is tropic for endometrial stromal and epithelial cells, leading to non-apoptotic cell death and *de novo *viral production associated with increased stromal cell prostaglandin-endoperoxide synthase 2 (PTGS2) protein and prostaglandin E_2 _(PGE) secretion [11,12]. The successfull replication of BoHV-4 in bovine endometrial cells was attribuited to post-entry events, with rapid viral reconstitution following the electroporation of nude viral DNA into endometrial stromal and epithelial cells [11,12]. A plausible mechanism underling this rapid activation of BoHV-4 replication in the endometrium is the capability of endometrial cells to transactivate the BoHV-4 Immediate Early 2 (IE2) gene promoter [11]. The IE2 gene is the molecular master swich for herpesvirus replication [13]. Furthermore, extracellular stimuli associated with the intrauterine microenvironment such as *E. coli *LPS and PGE transactivated the BoHV-4 IE2 gene promoter and viral replication [11]. BoHV-4 replication was also reactivated in latently infected macrophages when cocultured with endometrial stromal cells [11,12]. Thus, a model for endometrial BoHV-4 disease was proposed [1], involving a vicious circle comprising of bacterial endometritis leading to secretion of PGE, then PGE and LPS stimulating viral replication, which causes further endometrial tissue damage and inflammation.

Although BoHV-4 has been isolated from different lesions and from healthy animals, the relationship between biotypes of BoHV-4 and uterine disease has not been explored. The present study aimed to develop a tool enabling precise genetic discrimination between strains of BoHV-4 and to easily manipulate the viral genome. BoHV-4 was isolated from a cow affected with non-responsive post-partum metritis, characterized and the genome cloned as a bacterial artificial chromosome (BAC). This new uterine BAC-BoHV-4 clone represents a resource for functional genomic studies of BoHV-4 genes adapted to the endometrium, and will lead to new insights into the relationship between BoHV-4 and postpartum metritis.

## Methods

### Herd screening and isolation of BoHV-4

A dairy herd comprising of 73 cows that had a high incidence of postpartum metritis, abortion, and infertility was screened by indirect fluorescent antibody test (IFAT) for BoHV-4 antibodies. Briefly, IFAT was performed as follow: BoHV-4 infected and uninfected BEK cells were seeded onto 18-well glass slides, fixed in cold acetone and stored at -20°C until used. Sera were serially diluted in phosphate buffered saline (PBS) and 30 μl/well added to the infected and uninfected cells. For each slide, negative and positive control sera were used, as well as control wells containing BoHV-4 infected or uninfected cells treated only with the secondary antibody. After 1 h incubation at 37°C, the slides were washed three times in PBS and incubated for 1 h at 37°C with fluorescein isothiocyanate conjugated anti-bovine immunoglobulin G. At the same time, cells were counterstained with Evans Blue Dye (EBD; Sigma). Fluorescence was detected using a Zeiss Axiovert epifluorescent microscope and images acquired with the Axiocam Zeiss system. Sera were considered positive for BoHV-4 antibody when green fluorescence was detected at a dilution ≥ 1:40, with no fluorescence for uninfected cells at 1: 20.

### Endometrial cells collection by uterine trypsin flushing

A Holstein Friesian cow with a history of metritis was identified by IFAT as persistently infected with BoHV-4. The cow was 4 years old with a body condition score of 3 (scale 1 to 5), with no pyrexia or other clinical signs of disease. The cow was treated with a prostaglandin F_2α _analogue (Striate, Schering-Plough, Milano, Italy) to induce estrus 4 days later when endometrial cells were collected by flushing the uterus. Briefly, a Foley catheter was introduced through the cervix into the uterine lumen guided by rectal palpation and 50 ml of a trypsin EDTA solution (0,25% trypsin, 1 mM EDTA in PBS) infused into the lumen and the uterus gently massaged for 2 min before aspiration into a 50 ml tube. After collection, 5 ml FBS was added to the suspension and the endometrial cells were collected by centrifugation at 500 × g for 5 min. The cells were washed twice with 10 ml medium comprising of Dulbecco's modified essential medium (D-MEM; Sigma), 10% FBS (Sigma), 50 IU/ml penicillin (Sigma), 50 μg/ml streptomycin (Sigma) and 2.5 μg/ml Amphotericin B (Sigma), and then re-suspended in 2 ml of medium and stored at -80°C.

### BoHV-4-U isolation

The following BoHV-4-sensitive cell lines were obtained: bovine arterial endothelial (BAE-7372) from S. Grolli (Veterinary Biochemistry Institute, Parma University, Italy); Madin Derby Bovine Kidney (MDBK CCL-22) from ATCC (ATCC American Type Culture Collection, Manassas, USA); and bovine embryo kidney (BEK) and bovine embryo lung (BEL) cells from M. Ferrari (Istituto Zooprofilattico Sperimentale, Brescia, Italy). All cells were cultured in D-MEM (Sigma) containing 10% FBS (Sigma), 2 mM of L-glutamine (Sigma), 100 IU/ml of penicillin (Sigma), 100 μg/ml of streptomycin (Sigma) and 2.5 μg/ml of Amphotericin B (Sigma). The uterine endometrial cells were co-cultured with BAE-7372, MDBK, BEL and BEK cells in culture flasks until plaques characteristic of a cytopathic effect (CPE) developed. Single plaques were purified using 0.2% agarose (Sigma) gel three times. Flasks containing plaques were frozen in liquid nitrogen and thawed, the medium containing cell plaques centrifuged at 500 × g and the supernatant filtered through a 0.2 μm syringe filter (Minisart, Sartorius) and stored at -80°C.

### BoHV-4-U plaque immuno-peroxidase staining

The cell plaques were washed three times with PBS (Sigma) and fixed in 4% paraformaldehyde (Sigma) for 10 min at 37°C. The cells were washed twice and incubated for 5 min with PBS, 0.1% BSA at 20°C, and then incubated for a further 5 min with PBS, 0.3% Triton X-100 (Sigma). The cells were then washed three times with PBS, and incubated for 10 min at 37°C with 0.15% hydrogen peroxide (Sigma) in PBS. Rabbit anti-BoHV-4 hyperimmune serum, diluted 1:500 in PBS, was then added to the cells for 2 h at 37°C. After three washes with PBS, the cells were incubated with peroxidase-conjugated secondary antibody (Sigma), diluted 1:500 in PBS, for 1 h at 37°C, and washed three times with PBS. Secondary antibody was detected by development in 250 μg/ml DAB (Sigma), 0.015% H_2_O_2_, 50 mM Tris pH 7.4, for 10 min at 20°C.

### BoHV-4-U characterization

Viral infected cells were lysed overnight in TEL (10 mM Tris-HCl, pH 7.5, 1 mM EDTA) buffer containing 0.5% sodium dodecyl sulfate (SDS) (Sigma) and 100 μg/ml of proteinase K (Sigma) at 37°C. Nucleic acids were extracted by treatment with phenol-chloroform and precipitated with ethanol. Treatment with 100 μg/ml of RNAse A (Sigma) was performed for 1 h, after which the DNA was extracted with phenol and precipitated with ethanol again. The samples were kept at -20°C. One microgram of sample DNA was amplified over 30 cycles, each cycle consisting of denaturation at 94°C for 1 min, primer annealing at 55°C for 1 min, and chain elongation with 1 U of Taq polymerase (Roche) at 72°C for 2 min. PCR amplification was carried out in a final volume of 50 μl of 10 mM Tris-hydrochloride pH 8.3 containing 0.2 mM deoxynucleotide triphosphates (Roche), 3 mM MgCl_2 _(Roche), 50 mM KCl (Sigma) and 0.25 μM of each primer (MWG, Germany). In the first cycle, the samples were denatured at 94°C for 5 min, and in the last cycle the extension step was increased to 7 min. The primers used for amplification were selected from the published sequence of BoHV-4 genome [14] (sense primer:5'-cgaattctagtctaaagtcatcctc-3'; antisense primer: 5'-cgaattccattggcttcatcccaca-3'. Accession number: NC_002665). The PCR products were electrophoresed in 1% agarose gel and visualized after ethidium bromide staining. The amplified 2538-bp TK fragment was extracted from the agarose gel, digested with HindIII restriction endonuclease, and analyzed on 1.5% agarose gel in 1× TAE buffer (40 mM Tris-acetate, 1 mM EDTA) containing ethidium bromide. The specificity of the PCR product was determined by sequencing and compared with the GeneBank deposed sequence, Accession number: S49773 or NC_002665. Sequence alignment was performed with: Basic Local Alignment Search Tool (NCBI, National Center for Biotechnology Information, USA)

### RNA extraction and RT-PCR

Total RNA from infected and uninfected cells was extracted with TriPure reagent (Roche) and 2 μg of total RNA were reverse transcribed using Ready-To-Go, T-Primed First-Strand Kit (Amersham Biosciences, Milano Italy). Three microliters of reverse transcribed RNA were amplified over 30 cycles, each cycle consisting of denaturation at 94°C for 1 min, primer annealing at 55°C for 1 min, and chain elongation with 1 U of Taq polymerase (Boehringer-Diagnostics, Milano, Italy) at 72°C for 2 min. PCR amplification was carried out in a final volume of 50 μl of 10 mM Tris-hydrochloride, pH 8.3, containing 0.2 mM deoxynucleotide triphosphates, 3 mM MgCl_2_, 50 mM KCl and 0.25 μM of each primer (sense primer: 5'-acaaacacacagaccagtca-3'; antisense primer: 5'-gtttcacaacagattgagca-3' Primers were selected from the published sequence of BoHV-4 genome. Accession Number NC002665) [14].

### BAC-BoHV-4-U generation

For BAC cloning of the BoHV-4-U genome, a strategy employed to BAC clone the BoHV-4-A genome was used [15]. The intergenic region between the ORF2 and ORF3 was chosen because it was previously shown to be suitable for the introduction of the BAC cassette, and pSP72Bo2-*lox*GFP-BAC*lox*-Bo3 was excised from pSP72Bo2-*lox*GFP-BAC*lox*-Bo3 [15] by PvuII/ClaI restriction digestion and the gel-purified fragment was co-electroporated with 1 μg of purified BoHV-4-U DNA in BEK cells. GFP positive plaques were isolated and transferred onto newly prepared BEK cells. After several rounds of isolation, circular viral DNA intermediates were isolated from infected cells by the Hirt isolation method [16] and transferred into *Escherichia coli *DH10B (Promega, Milano, Italy). The transformants were plated on agar containing 17 μg/ml chloramphenicol. DNA was extracted from each positive clone, restriction enzyme digested, and analyzed by electrophoresis on 1% agarose gel. DNA fragments were visualized with a UV transilluminator.

### Cell culture electroporation and recombinant virus reconstitution

BEK or BEK*cre *cells were maintained as a monolayer with complete medium containing 90% D-MEM, 10% FBS, 2 mM l-glutamine, 100 IU/ml penicillin and 10 μg/ml streptomycin. Cells were sub-cultured in a fresh culture vessel when growth reached 70–90% confluence (i.e., every 3–5 days) and incubated at 37°C in a humidified atmosphere with 5% CO_2_. BAC-BoHV-4 plasmid DNA (5 μg) in 500 μl D-MEM without serum was electroporated (Equibio apparatus, 270 V, 960 μF, 4-mm gap cuvettes) into BEK or BEK*cre *cells from a confluent 25-cm^2 ^flask. Electroporated cells were returned to the flask, fed the next day, and split 1:2 when they reached confluence 2 days after electroporation. Cells were left to grow until CPE appeared. Recombinant viruses were propagated by infecting confluent monolayers of BEK or MDBK cells at a m.o.i. of 0.5 TCID_50 _per cell and maintaining in MEM with 10% FBS for 2 h. The medium was then removed and replaced by fresh MEM containing 10% FBS. When approximately 90% of the cell monolayer exhibited CPE (approximately 72 h post-infection), the virus was prepared by freezing and thawing cells three times and pelleting virions through 30% sucrose. Virus pellets were resuspended in ice cold D-MEM without FBS. TCID_50 _were determined on MDBK cells by limiting dilution.

### BAC-BoHV-4-U genome modification by recombineering

Recombineering was performed as described by Warming et al. (2005) [17] with some modifications. Briefly, 500 μl SW102 *E. coli *containing BAC-BoHV-4-U cultured overnight at 32°C was diluted in 25 ml Luria-Bertani (LB) (Sigma) medium with or without 12.5 μg/ml chloramphenicol (Sigma) selection and grown at 32°C in a 50 ml baffled conical flask to an OD_600 _of 0.6. Then, 10 ml were transferred to another baffled 50 ml conical flask and heat-shocked at 42°C for exactly 15 min in a shaking water bath. The remaining culture was left at 32°C as the uninduced control. After 15 min the two samples were briefly cooled in an ice/water bath, transferred to 15 ml Falcon tubes and pelleted at 3000 × g for 5 min at 0°C. The supernatant was poured off and the pellet was resuspended in 1 ml ice-cold ddH_2_O by gently swirling the tubes in an ice/water bath. Subsequently, 9 ml ice-cold ddH_2_O were added and the samples pelleted again. This step was repeated once more, the supernatant was removed and the pellet (50 μl each) was kept on ice until electroporated with a gel-purified ~4.3 kb fragment (TK-KanaGalK-TK) obtained by cutting pTK-KanaGalK-TK with XhoI/EcoRI (Roche). An 25 μl aliquot was used for each electroporation (Equibios apparatus, Equibios, Milano, Italy) in a 0.1 cm cuvette (Promega) at 25 μF, 2.-5 kV and 201 Ω. After electroporation, the bacteria were recovered in 1 ml LB (15 ml Falcon tube) for 1 h in a 32°C shaking water bath. For the counter selection step (see below), the bacteria were recovered in 10 ml LB in a 50 ml baffled conical flask and incubated for 4.5 h in a 32°C shaking water bath.

After the recovery period, the bacteria were washed twice in sterile 1× M9 salts (6 g/l Na_2_HPO_4_, 3 g/l KH_2_PO_4_, 1 g/l NH_4_Cl and 0.5 g/l NaCl,) (Sigma) as follows: 1 ml culture was pelleted in an eppendorf tube at 10,000 × g for 15 min and the supernatant was removed with a pipette. The pellet was resuspended in 1 ml of 1× M9 salts, and pelleted again. This washing step was repeated once more. After the second wash, the supernatant was removed and the pellet was resuspended in 1 ml of 1× M9 salts before plating serial dilutions (100 μl each of 1:10, 1:100 and 1:1000 dilutions) on M63 minimal medium plates (agar 15 g/l; (DIFCO, BD Biosciences, Milano, Italy), 0.2% D-galactose (Sigma), 1 mg/l D-biotin (Sigma), 45 mg/l L-leucine (Sigma) and 50 mg/l kanamycin (Sigma). Washing in M9 salts is necessary to remove any rich media from the bacteria prior to selection on minimal medium plates. Plates were incubated for 3 to 5 days at 32°C. Several selected colonies were picked and streaked on McConkey agar indicator plates (DIFCO, BD Biosciences) containing 50 μg/ml of kanamycin, and incubated at 32°C for 3 days until red colonies appeared. Red colonies were grown overnight in 5 ml of LB containing 50 μg/ml of kanamycin and BAC-BoHV-4-U was purified and analyzed through HindIII restriction enzyme digestion for TK-KanaGalK-TK fragment targeted integration into the BoHV-4 TK locus.

## Results

### BoHV-4 isolation from the uterus of an infected animal

**Figure 1:**
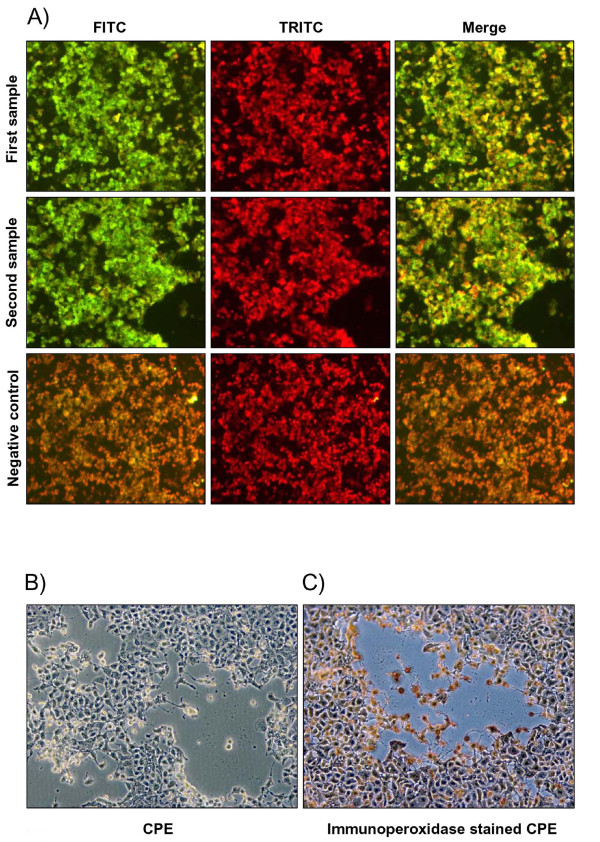
**A) Representative microscopic (10×) IFAT images of sera from the cow which was used to isolate BoHV-4-U**. The presence of anti-BoHV-4 antibodies in the first and second sample (6 months following the first) are detectable by green cells when observed with a FITC filter and the negative controls appears brown due to the Evans blue dye. Counterstaining with Evans blue dye observed with a TRITC/filter (red pictures) was used to monitor the integrity of the cell substrate. The merged pictures (FITC/TRITC) allow co-localization of the anti-BoHV-4 antibody staining with the cellular substrate. **B) **Representative phase contrast microscopic images (10×) of BoHV-4-U cytopathic effect generating plaques. **C) **Representative phase contrast microscopic images (10×) of BoHV-4-U plaques immuno-peroxidase stained with anti-BoHV-4 antibodies.

A cow with a history of postpartum metritis was BoHV-4 IFAT positive twice at 6-month intervals (Fig. 1A). Uterine cells were collected from this animal and co-cultivated with BoHV-4 permissive cell lines. Ten days after co-culture and one cell passage, a CPE typical of BoHV-4 appeared in the BEK cells (Fig. 1B). The presence of BoHV-4 was confirmed by immuno-peroxidase staining of the plaques with anti-BoHV-4 antibodies (Fig. 1C).

### BoHV-4 isolate characterization

**Figure 2:**
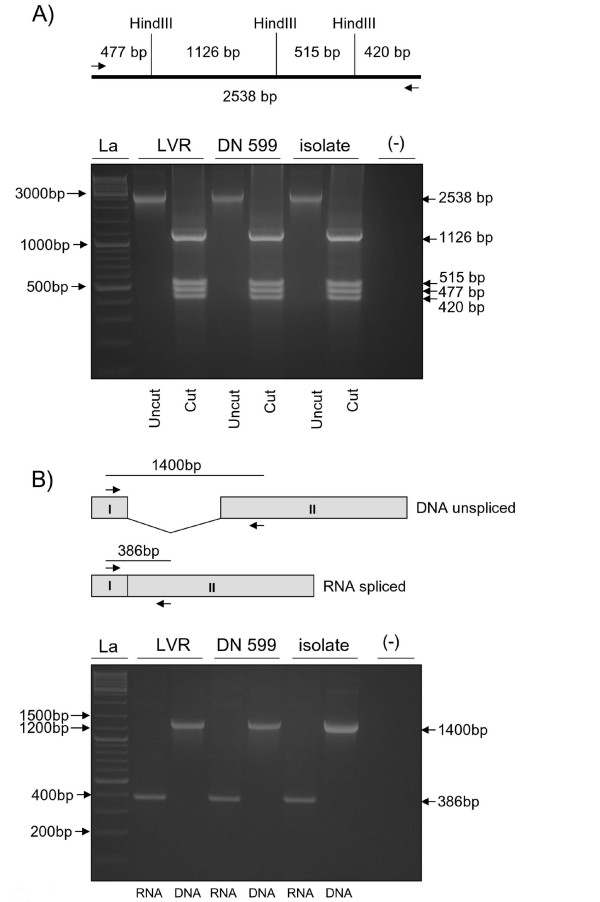
**A) Predicted location of *Hin*dIII restriction sites and respective expected restriction fragment sizes (not shown to scale)**. Ethidium bromide stained agarose gel electrophoresis of specific amplification of BoHV-4 DNA fragment from LVR, DN599 reference strains and the BoHV-4-U isolate. PCR amplified 2,538-bp fragment (Uncut) and digested with HindIII restriction enzyme (Cut). Lanes (-) correspond to negative controls and La to 1 kb ladder molecular size marker. **B) **Diagram showing the genomic region containing the BoHV-4 IE2 gene (not to scale), where the exon II contains most of the IE2 ORF, except the translation initiation codon contained into the exon I. During splicing, an intron is removed and the two exons (I and II) are joined together to generate the IE2 ORF. The position of the primers used to amplify the region containing IE2 are shown by arrows. The selected primers generate an amplicon of 1400 bp from the viral IE2 genomic region, while the amplicon is only 386 bp in length from the spliced product of IE2 transcript, thus allowing distinction between viral genomic DNA and the reverse transcribed IE2 RNA. Ethidium bromide stained agarose gel electrophoresis of RT-PCR or PCR of RNA and DNA from LVR, DN599 reference strains and the new isolate BoHV-4-U infected cells.

To confirm the specificity of the BoHV-4 isolate, single-plaque purified virus was analyzed by RFLP-PCR of the TK locus (Fig. 2A) and RT-PCR for the spliced form of the IE2 gene (Fig. 2B), which is a specific marker for BoHV-4 immediate early gene expression [13]. Both methods confirmed the specificity of the uterine isolate, which was called BoHV-4-U. A restriction profile comparing the BoHV-4-U viral genome with reference strains (LVR and Movar, kindly provided by Professor Castrucci, University of Perugia, Pisa Italy. DN 599, ATCC-VR-631), classified the isolate as Movar-like (data not shown), which was in agreement with the geographical distribution of the Movar strain in the north of Italy [18].

### Cloning of BoHV-4-U as a bacterial artificial chromosome

**Figure 3:**
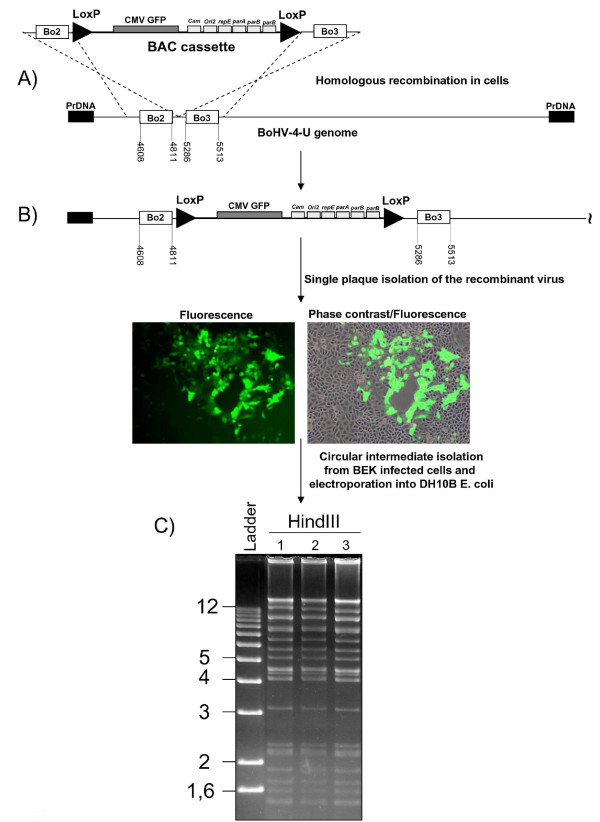
**Representative flow chart of the strategy used to generate pBAC-BoHV-4-U**. **A) **Through classical homologous recombination in eukaryotic cells obtained by co-transfection, the floxed (*loxP *sites indicated by triangles) CMV-GFP BAC cassette containing F1 plasmids elements (chloramphenicol resistant gene, Cam; F1 origin of replication, Ori2; partitioning protein genes, repE, parA, parB and parC), flanked by two homologous BoHV-4-U regions, Bo2 and Bo3, was introduced into the intergenic region of the BoHV-4-U genome [Numbers indicates sequence location in BoHV-4 genome based on the Zimmermann's sequence [14]]. **B) **Diagram of the recombinant BoHV-4-U and images of a single plaque expressing GFP (Fluorescence and phase contrast merged on fluorescence. 10×). **C) **Ethidium bromide stained agarose gel of three (1, 2, 3) representative HindIII digested pBAC-BoHV-4-U clones.

To facilitate further characterization and manipulation of BoHV-4-U, the genome was cloned as a bacterial artificial chromosome. The intergenic region located between Bo2 and Bo3 ORFs (situated to the left end of the viral genome, Fig. 3A) [14] was selected as a target site to introduce the BAC cassette. Plasmid (pBo2-EGFP-BAC-Bo3), containing homologous regions flanking the floxed BAC cassette for *cre *excision and an EGFP expression cassette, was linearized and co-transfected with BoHV-4-U purified genome into BEK cells. Recombinant virus was isolated from single plaques expressing EGFP and the structure of the recombinant virus was assessed by HindIII restriction profiling, PCR and sequencing. Circular intermediates of the recombinant BoHV-4-U were isolated from newly infected BEK cells and electroporated into *E. coli *DH 10B to generate pBAC-BoHV-4-U (Fig. 3B). HindIII restriction profile (Fig. 3C), Southern blotting, PCR and partial sequencing (data not shown) confirmed the integrity of the clone.

### Clonal stability and viral reconstitution

**Figure 4:**
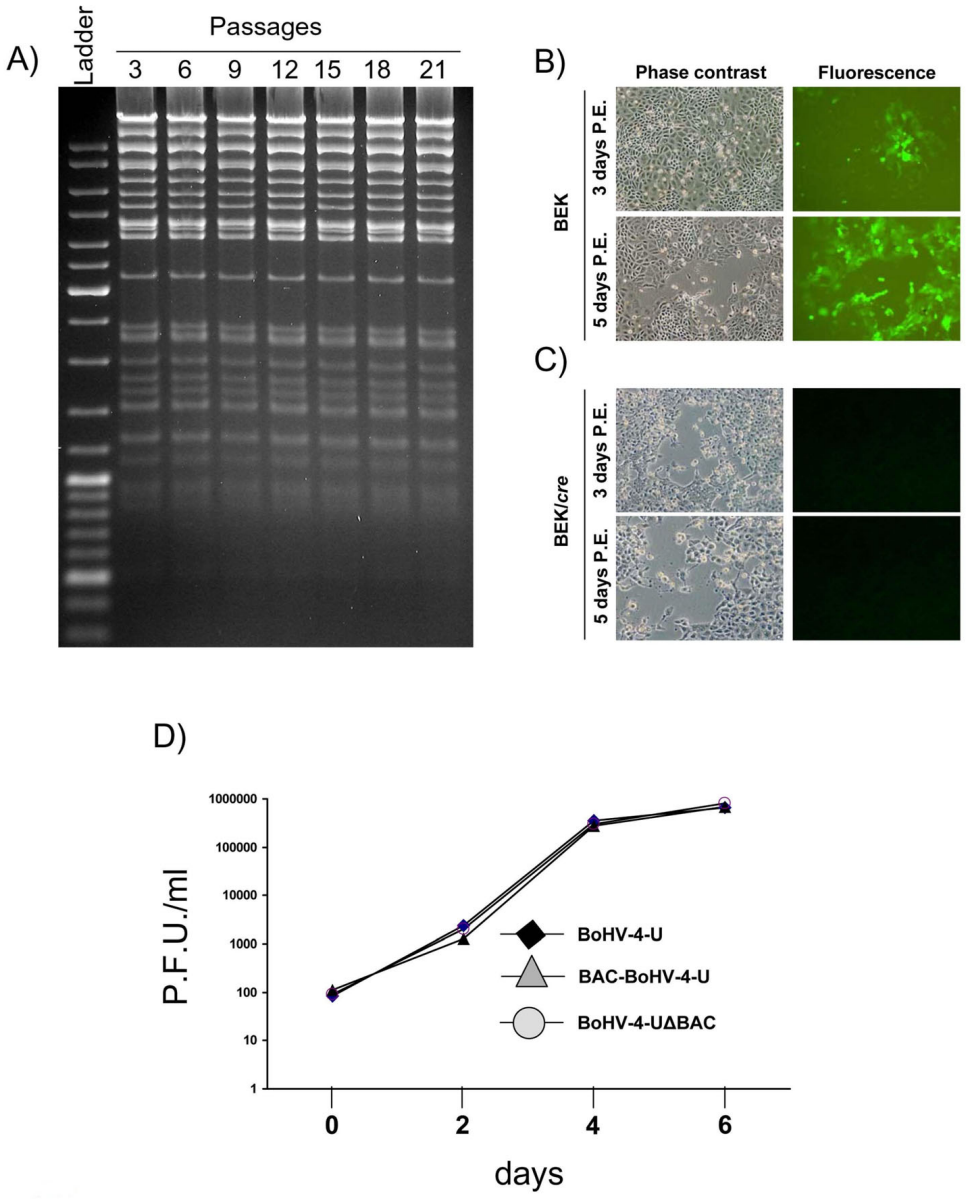
**A) Stability of the pBAC-BoHV-4-U plasmid in *E. coli *DH10B**. DH10B containing the pBAC-BoHV-4-U were passaged for 21 consecutive days, BAC DNA from the culture was prepared on the indicated days (3, 6, 9, 12,15, 18 and 21), HindIII digested and analyzed on ethidium bromide stained agarose gel. **B) **BEK or **C) **BEK expressing *cre *cells (BEK/*cre*) at 3 and 5 days post electroporation with pBAC-BoHV-4-U, where BAC-BoHV-4-U virus is reconstituted causing CPE and spreading through the cellular monolayer as shown by both phase contrast and fluorescence images (in B). Whereas, due to the expression of *cre *and the BAC cassette containing GFP is removed from the BAC-BoHV-4-U reconstituted, CPE is appreciable in phase contrast images but not in fluorescence (in C). **D) **Replication kinetics of BoHV-4-A-U reconstituted virus in BEK/cre cells (the BAC cassette has been removed by cre), compared with those of the parental BAC-BoHV-4-U (virus reconstituted on un-expressing cre BEK cells) still containing the BAC cassette and the BoHV-4-U isolate. The data presented are the means ± standard errors of triplicate measurements, P > 0,05 for all time points as measured by Student's *t *test.

The stability of the pBAC-BoHV-4-U clone in bacteria was confirmed over twenty passages (Fig. 4A). Electroporation of the pBAC-BoHV-4-U plasmid into BEK cells, allowed infectious virus reconstitution as observed by plaques expressing green fluorescent protein formation and CPE spreading through the cell monolayer over time (Fig. 4B). The BAC cassette was successfully excised from the BAC-BoHV-4-U, as monitored by the lost of GFP expression, when the BAC-BoHV-4-U virus was growth on BEK cells expressing *cre *or the pBAC-BoHV-4-U plasmid was electroporated into BEK cells expressing *cre *(Fig. 4C). There were no differences in growth characteristics between BoHV-4-U, BAC-BoHV-4-U and BoHV-4-UΔBAC (Fig. 4D).

### Insertional mutagenesis into BoHV-4-U genome cloned as Bacterial Artificial Chromosome

**Figure 5:**
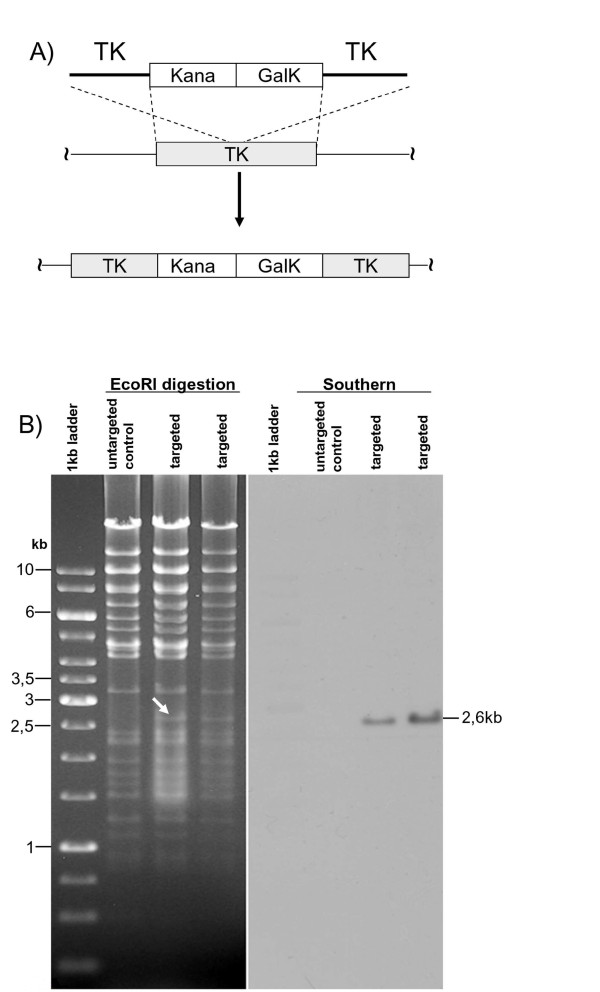
**(A) Kanamycin resistant cassette (Kana) adjacent to the galactokinase cassette (GalK) and flanked by BoHV-4 thymidine Kinase gene and adjacent sequences (TK), are introduced into the TK gene of BoHV-4-U genome cloned as bacterial artificial chromosome (BAC-BoHV-4-U) via heat inducible homologous recombination in SW 102 *E. coli***. Following a positive selection on (solid) minimal plates containing galactose as the only source of carbon and a positive selection on (liquid) medium containing Kanamycin. **b) **HindIII restriction enzyme analysis of 2 clones out of hundreds, displaying the right targeting, where a new 2.6 kb band (indicated by arrow) corresponding to the insertion of the Kana/GalK cassettes is present but missing in the untargeted control. Southern hybridization with a Kanamycin specific probe confirms the specific targeting.

The BAC recombineering system [17], modified by the introduction of a kanamycin selection step, was used to test the feasibility of using BoHV-4-U for mutagenesis. The galactokinase cassette (*Gal*K) along with a kanamycin resistance cassette, both under the control of constitutively active prokaryotic promoters, were subcloned in pINT2 [19] to be flanked by the BoHV-4 TK gene and adjacent sequences. The generated targeting vector (TK-KanaGalK-TK) was excised out from the plasmid backbone and used for heat-inducible homologous recombination in SW102 *E. coli *containing the BAC BoHV-4 genome. Following positive selection in minimal medium containing galactose and a second positive selection with medium containing kanamycin, all the clones analyzed displayed the correct target (Fig. 5).

## Discussion and conclusion

BoHV-4 is a herpesvirus belonging to the gammaherpesvirus family, genus *Rhadinovirus *[14]. Although BoHV-4 has been isolated from animals with various clinical symptoms and from apparently healthy animals, BoHV-4 isolation from animals with disease of the female genital tract is the best documented and one of the few cases where disease could be reproduced [20]. Wellemans et al, (1986) reproduced metritis by infecting cows at various times before parturition; no clinical signs were seen after primary infection but metritis was observed after parturition. It is likely that BoHV-4 causes post-partum metritis along with other microbes such as the bacteria *E. coli *and *A. pyogenes *[11]. In the present study a BoHV-4 strain (BoHV-4-U) was isolated from the uterus of a persistently infected cow, affected by postpartum metritis. The authenticity of the isolate was confirmed by RFLP-PCR and sequencing using a large 2,538 bp conserved region (TK lokus) as a genetic marker region for the BoHV-4 genome, [21], including the 3' end of ORF1 (homologous to the EBV BVRF1 gene), ORF2 (homologous to the EBV BXRF1 gene), ORF3 (TK gene) and ORF4 (gH gene) 5' end. The presence of the spliced form of the IE2 gene, a specific marker for BoHV-4 immediate early expression [22], was identified by RT-PCR and sequencing. The BoHV-4-U genome was cloned as a bacterial artificial chromosome (BAC) and the stability of the pBAC-BoHV-4-U clone was confirmed over twenty passages. Furthermore, the growth of the engineered virus was similar to the growth of the wild type virus. Finally, the feasibility of using BoHV-4-U for mutagenesis was demonstrated using the BAC recombineering system.

Strain adaptation of microbes for an organ, tissue or cell type is an important issue for the pathogenesis of disease. Differences observed in the disease potential of BoHV-4 may constitute an adaptation of the virus to different selection pressures that increase the frequency of the most pathogenic genetic variants. These selection pressures might include the endometrial environment, so to understand the effects of BoHV-4 on uterine function it is important to use a specific BoHV-4 isolated from a case of postpartum metritis. For example, Nuget et al. (2006) [23] found that neuro-pathogenic EHV-1 strains encode G whereas non-pathogenic EHV-1 strains encode A in position 2,254 of the genome corresponding to the viral DNA polymerase (*pol*) ORF. The relationship between polymorphism in EHV-1 *pol *and neuro-pathogenicity was verified by targeted mutagenesis of a single nucleotide in the DNA genome of the virus cloned as bacterial artificial chromosome [24]. Similarly, the uterine isolate of BoHV-4 genome cloned as bacterial artificial chromosome could then be modified by homologous recombination and the virus reconstituted in eukaryotic cells following transfection to study mechanisms of pathogenicity.

The primary method for investigating the function of individual herpesvirus genes is mutagenesis. Mutated viruses are usually constructed by homologous recombination following the co-transfection of viral genomic DNA and a mutated allele on a separate DNA fragment [25]. Recombinant viruses are either screened or selected during several sequential rounds of plaque purification. However, if the mutation results in growth defects relative to wild-type parental virus, the mutant virus may be difficult or impossible to purify. Lethal mutations are often detected only indirectly by the unsuccessful isolation of the desired mutant virus. Such mutants can be isolated and confirmed as lethal only by the construction of a complementary cell line expressing the wild-type allele. To overcome these limitations, many herpesvirus have been cloned as an infectious bacterial artificial chromosome (BAC).

Although the genome of BoHV-4 has been sequenced completely [14] the function of most of the gene remains unknown. Fewer than 75 ORFs have been characterized in BoHV-4, based on amino acids homology and genome location. While most of the genes have no apparent homology to known viral or cellular genes, some are important for virus-host interaction. For example, IE2 gene, is the molecular master switch for BoHV-4 replication, and the capability of endometrial cells to transactivate the IE2 promoter was previously investigated by transient transfection with a reporter construct containing the IE2 promoter transfected into endometrial cells [11]. Therefore the mutation of the IE2 gene in BoHV-4-U would be of great interest, to understand its function in the context of the interaction between a specific uterine strain (BoHV-4-U) and the endometrial cells.

The functional analysis of BoHV-4-U genes in endometrial cells is a fundamental prerequisite for regulatory region discovery. Gene trap technology [26] in combination with transposition technology, site direct mutagenesis and homologous recombination on BAC-BoHV-4-U genome, will provide an important tool to carry out a large-scale search and analysis of BoHV-4-U regulatory regions. Targeting genes or regions of gene expression regulation and the phenotypic analysis of the BoHV-4-U mutants, represents a powerful tool to study the direct and indirect interactions of genes and genomic elements in a biological context, such as in the uterus. The classification of mutants into functional clusters will help identify uncharacterized genes responsible for BoHV-4 strain adaptation to the uterine microenvironment. The established BoHV-4-U library of mutants together with different endometrial cell types will cover a substantial amount of biological information of intrinsic value about the functional relationship of the targeted genes and the host background.

In conclusion, the uterine adapted BoHV-4-U genome cloned as a bacterial artificial chromosome provides a tractable tool that can be used to explore the complex interactions between BoHV-4 and the host cells of the endometrium.

## Competing interests

The authors declare that they have no competing interests.

## Authors' contributions

GD conceived, designed, performed the experiments and wrote the paper. VF and AC contributed to perform the experiments. IMS intellectually contributed and helped to write the paper. All authors read and approved the final manuscript.
